# Refractive error and axial length prediction using fundus parameters of normal eyes in the Kumejima population study

**DOI:** 10.1007/s10384-025-01192-5

**Published:** 2025-06-09

**Authors:** Takehiro Yamashita, Ryo Asaoka, Aiko Iwase, Hiroshi Sakai, Hiroto Terasaki, Taiji Sakamoto, Makoto Araie

**Affiliations:** 1 Department of Ophthalmology, Kagoshima University Graduate School of Medical and Dental Sciences, Kagoshima, Japan; 2 Department of Ophthalmology, Seirei Hamamatsu General Hospital, Shizuoka, Japan; 3 Tajimi Iwase Eye Clinic, Gifu, Japan; 4 Urasoe Sakai Eye Clinic, Okinawa, Japan; 5 Department of Ophthalmology, Kanto Central Hospital, Tokyo, Japan

**Keywords:** Color fundus photographs, Axial length, Refractive error, Population study, Fundus parameters

## Abstract

**Purpose:**

To evaluate the accuracy of predictive refractive error (RE) and axial length (AL) using regression analysis of fundus parameters in the Kumejima study and to identify RE- or AL-related fundus changes.

**Study Design:**

Prospective cross-sectional observational population study.

**Methods:**

Non-mydriatic color fundus photographs (CFPs) from 1,646 right eyes of healthy Kumejima participants were analyzed. Mean red (R), green (G), and blue (B) intensities at eight locations around the optic disc and fovea were quantified, and the tessellation fundus index was calculated as R/(R + G + B). Optic disc ovality ratio, papillomacular angle, and retinal vessel angle were measured. Least absolute shrinkage and selection operator regression with leave-one-out cross-validation predicted RE and AL, validated using Pearson’s correlation coefficient.

**Results:**

The mean ± standard deviation actual RE and AL of participants (834 men and 812 women) were -0.14±1.62 diopter and 23.50±0.88 mm. The mean ± standard deviation predicted RE and AL based on fundus parameters was -0.14±1.05 diopter and 23.50±0.48 mm, with a mean absolute error of 0.91 diopter and 0.59 mm, and the correlation coefficients between actual and predicted RE and AL were 0.63 and 0.51 (p<0.001). Eyes with a longer AL had narrower temporal vessel angles, weaker green intensities, stronger blue intensities, and increased tessellation of the fundus color (p<0.001).

**Conclusion:**

RE and AL could be predicted using CFP parameters; the RE- or AL-related changes in the fundus, such as vessel angles and peripapillary color intensity, may enhance our understanding of myopia mechanisms.

## Introduction

The ocular fundus, like the face, varies from person to person, and the variation is ample to be used for personal identification [1]. This implies that the fundus comprises several pieces of information that can help in identifying individuals. Artificial intelligence (AI), especially deep-learning AI, can estimate age, sex, systolic blood pressure, smoking habits, and the presence of cardiac complications [2]; refraction (RE) [3]; and axial length (AL) [4] using only fundus photographs. In particular, RE and AL can be estimated with a mean absolute error (MAE) within 0.56 diopter [3] and 0.56 mm [4]. However, it is impossible to identify the crucial factors for estimating the AL and RE of individuals despite using attentional heat maps or quantifying a few factors. Similarly, in chess, Go, and Shogi, deep-learning AI can predict the best moves or results; however, it is unknown why the predicted move is superior [5], whereas professional chess players need to study the reasons for AI moves. Consequently, it is challenging for professionals to explain the rationale behind the decisions made by deep-learning AI, referred to as black-box AI [6]. Furthermore, the complexity of the fundus surpasses these games, as the eye is a natural object.

Conventional statistical methods such as multiple regression analysis using various factors have been proposed as potential to unravel this black box [6]. Similar to the face, the fundus is unique and has many different features. For example, large individual variations exist in the angle or trajectory of the retinal vessels [7–11], location and shape of the optic disc [12, 13], and color of the peripapillary area [14–17]. The authors previously reported that regression with L2 regularization (ridge regression) using these fundus parameters could determine sex with an accuracy rate of 77.9% in young adults (persons in their 20s) [18], 63.2% in 8.5-year-olds [19], and 80.4% in a study population (>40 years old) [20]. Moreover, multiple regression analyses revealed sex-specific differences. The optic disc was oval-shaped, the retinal vessels were closer to the fovea, and the peripapillary color of the fundus appeared more greenish in women than in men [18–20].

We hypothesized that this method could predict AL and RE based on fundus parameters and reveal specific AL- or RE-related fundus changes. This study aimed to predict AL and RE in older individuals using regression analyses of color fundus photographs (CFPs). The CFPs of 1,646 eyes from individuals ≥40 years old who were participants in the Kumejima population study were investigated.

## Methods

### Study population

The study procedures conformed to the tenets of the Declaration of Helsinki and regulations of Japan, and the ethics committee of the town of Kumejima approved the study protocol. Written informed consent was obtained from all the participants before the examination.

The size of Kumejima Island is 63.2 km^2^, it is located in the southwestern part of Japan (eastern longitude of 126°48’ and northern latitude of 26°20’), west of the main island of Okinawa. With a population of approximately 9000, most of its residents originate from Okinawa Prefecture. This study was conducted between May 2005 and August 2006, and all residents who were ≥40 years were informed of the protocol and invited to participate. According to the official household registration database, Kumejima had 5249 residents aged ≥40 years in 2005. After excluding 617 residents who could not be examined for various reasons, 4632 were eligible for the study [21, 22].

### Examination and diagnosis

The screening examination comprised a structured interview, the details of which are published elsewhere [21, 22]. Ocular examinations were performed by experienced ophthalmologists and examiners, and best-corrected visual acuity, spherical equivalent (RE) (ARK-730, Topcon), intraocular pressure with a Goldmann applanation tonometer, and AL (IOLMaster, Carl Zeiss Meditec) were measured. Additionally, slit-lamp biomicroscopy, gonioscopy, ophthalmoscopy, fundus photography, and perimetry were performed. Sequential stereoscopic color fundus photographs (CFPs) were obtained at 45° using a non-mydriatic digital ocular fundus camera system (ImageNet TRC-NW7; Topcon). Details of the analyses of the optic disc, fundus, and visual field examinations and the diagnosis of ocular diseases are reported elsewhere [21, 22].

### Measurement of fundus parameters

**Fig. 1:**
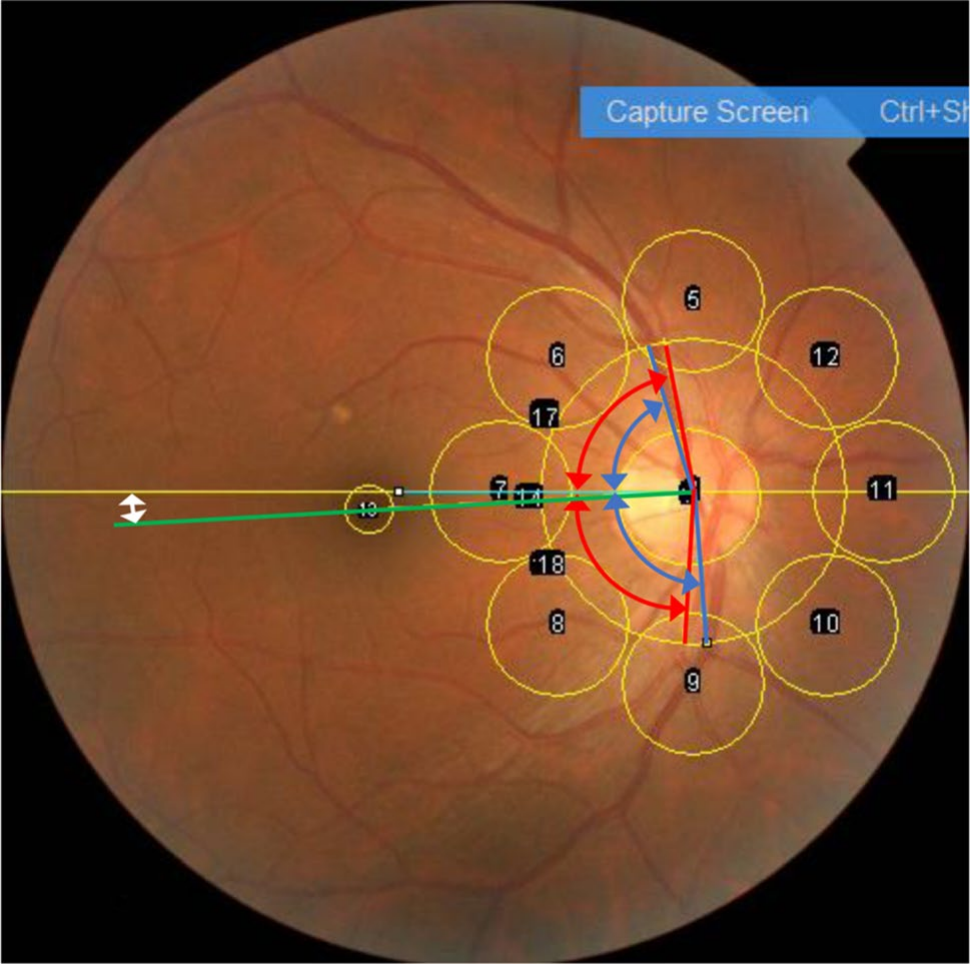
Method of quantifying retinal vessel angles and papillomacular position (PMP), ovality ratio, and red-green-blue intensity. Red double arrows indicate the supratemporal and infratemporal retinal artery angles (ST-RA and IT-RA). Blue double arrows indicate the supratemporal and infratemporal retinal vein angles (ST-RV, IT-RV). The white double arrow indicates the PMP. The ovality ratio was determined by dividing the maximum optic disc diameter by its minimum diameter. The red-green-blue intensities and tessellation fundus index (TFI) were calculated for each of the eight locations around the optic disc and fovea

As described in our previous study, 42 fundus parameters, including the supratemporal (ST) and infratemporal (IT) major retinal artery angles (RA) and retinal vein angle (VA) relative to the temporal horizontal line, were measured. These angles were named ST-RA, IT-RA, ST-VA, and IT-VA, as described previously [7, 8, 11, 20] (Fig. 1). The papillomacular position (PMP) was defined as the angle formed at the intersection of a horizontal line and a line connecting the optic disc center to the fovea [12].

The ovality ratio was calculated by dividing the maximum value by the minimum disc diameters [13]. The mean values of the red, green, and blue intensities within each area were calculated for eight foveal and peripapillary circle. The tessellation fundus index (TFI) was calculated using the mean red (R), green (G), and blue (B) intensities at each of the nine locations as follows: TFI = R/(R + G + B) [14, 15].

These measurements were performed using CFP images and ImageJ software (ImageJ version 1.47; National Institutes of Health; available at http://imagej.nih.gov/ij/). The macro function of ImageJ enabled the semi-automated calculation of these 42 CFP parameters when the locations of the fovea, edge of the optic nerve head, and crossing points of the supralateral and inferolateral RA or retinal veins were determined.

### Statistical analyses

As in our previous studies, the least absolute shrinkage and selection operator (LASSO) regression [23, 24] with 42 variables, including RGB intensities, TFI at nine locations, ST-RA, IT-RA, ST-VA, IT-VA, PMP, and ovality ratio, was used to predict AL and RE [25, 26]. LASSO regression with leave-one-out cross-validation was used to overcome the overfitting problem in ordinal statistical regression models, such as multivariate linear regression or binomial logistic regression, by applying a penalty to coefficients (L1 regularization), and the sum of the absolute values of the regression coefficients was regularized [23–26]. More precisely, let x ∈ R^p denote the variables and let y ∈R denote the response (please note x_ij are normalized and y has mean zero). The LASSO algorithm solves the following problem:

1 $$\mathop {\min }\limits_{{\left( {\beta 0, \beta } \right) \in R^{p + 1} }} \left[ {\frac{1}{2N}\mathop \sum \limits_{i = 1}^{N} \left( {yi - \beta 0 - x_{i}^{Y} \beta } \right)^{2} + \lambda P_{\alpha } \left( \beta \right)} \right],$$

2 $$P_{\alpha } \left( \beta \right) = \left( {1 - \alpha } \right)\frac{1}{2} \left| {\left| \beta \right||_{{l_{2} }}^{2} + \alpha } \right|\left| \beta \right||_{{l_{1} }}$$

3 $$= \mathop \sum \limits_{j = 1}^{p} \left[ {\frac{1}{2}{ }\left( {1 - {\upalpha }} \right)\beta_{j}^{2} + \alpha \left| {\beta_{j} } \right|} \right]$$

In equation (1), $\mathop {\min }\limits_{{\left( {\beta 0, \beta } \right) \in R^{p + 1} }} \left[ {\frac{1}{2N}\mathop \sum \limits_{i = 1}^{N} \left( {yi - \beta 0 - x_{i}^{Y} \beta } \right)^{2} } \right]$ is identical to OLSLR and $\lambda {P}_{\alpha }(\beta )$ is the penalty term for the shrinkage [23, 24].

The merit of this approach is that, unlike in deep learning, it is possible to directly observe the effects of the parameters in the optimal model, similar to ordinal multiple linear/logistic regression.

The diagnostic performance of the LASSO binomial logistic regression approach was evaluated using leave-one-out cross-validation. In the leave-one-out cross-validation, a single eye from the original dataset was used as validation data, and the remaining observations (1645 eyes) were used as training data [20, 27]. This procedure was repeated so that each eye was used once as validation data (1646 iterations). The diagnostic accuracy was evaluated using the area under the receiver operating characteristic (ROC) curve. The final optimal model was identified using all 1646 eyes.

The relationships between actual and predicted AL and RE were determined using Pearson’s correlation analysis, as were the relationships among AL, RE, and fundus parameters. The study sample size was relatively large; hence, the significance level was set at p<0.001. All statistical analyses were performed using SPSS Statistics version 19 for Windows (SPSS Inc., IBM) and R (ver. 3.1.3, R Foundation for Statistical Computing).

## Results

CFPs of acceptable quality were obtained from 7524 eyes of 3762 participants, with the presence or absence of fundus diseases determined. Photographs obtained from 376 right and 421 left eyes were excluded because of cataracts, corneal opacity, large pterygium, or small pupil. Eyes with prior intraocular surgery (453 OD, 443 OS) were excluded. Furthermore, eyes were excluded if the refractive error (RE) exceeded +5 diopters or was less than −8 (14 OD, 11 OS), or if optic disc diseases (including glaucoma), retinal diseases (including pathologic myopia), or brain diseases were present in either eye (711 OD, 679 OS). Finally, in 2208 individuals both eyes were judged to be acceptable. The detailed demographics of these eyes are explained in our earlier reports [20, 28, 29]. Only the right eye was used to measure the fundus parameters. Of the 2208 normal eyes, 562 were excluded because of the difficulty in measuring the fundus parameters due to the lack of clarity of the peripheral area of the fundus or unidentified fovea. Finally, 1646 right eyes were used for analysis. The demographic data of the right eyes of the eligible participants are listed in Table 1. All participants were >40 years of age.

**Table 1:** Participants data

	Mean ± standard deviation (range)
Number of eyes (male/female)	834/812
Age (years)	53.4±10.1 (40–80)
Refractive error (diopters)	– 0.14±1.62 ( – 7.625 to 5.500)
Axial length (mm)	23.50±0.88 (20.92–27.55)

**Fig. 2:**
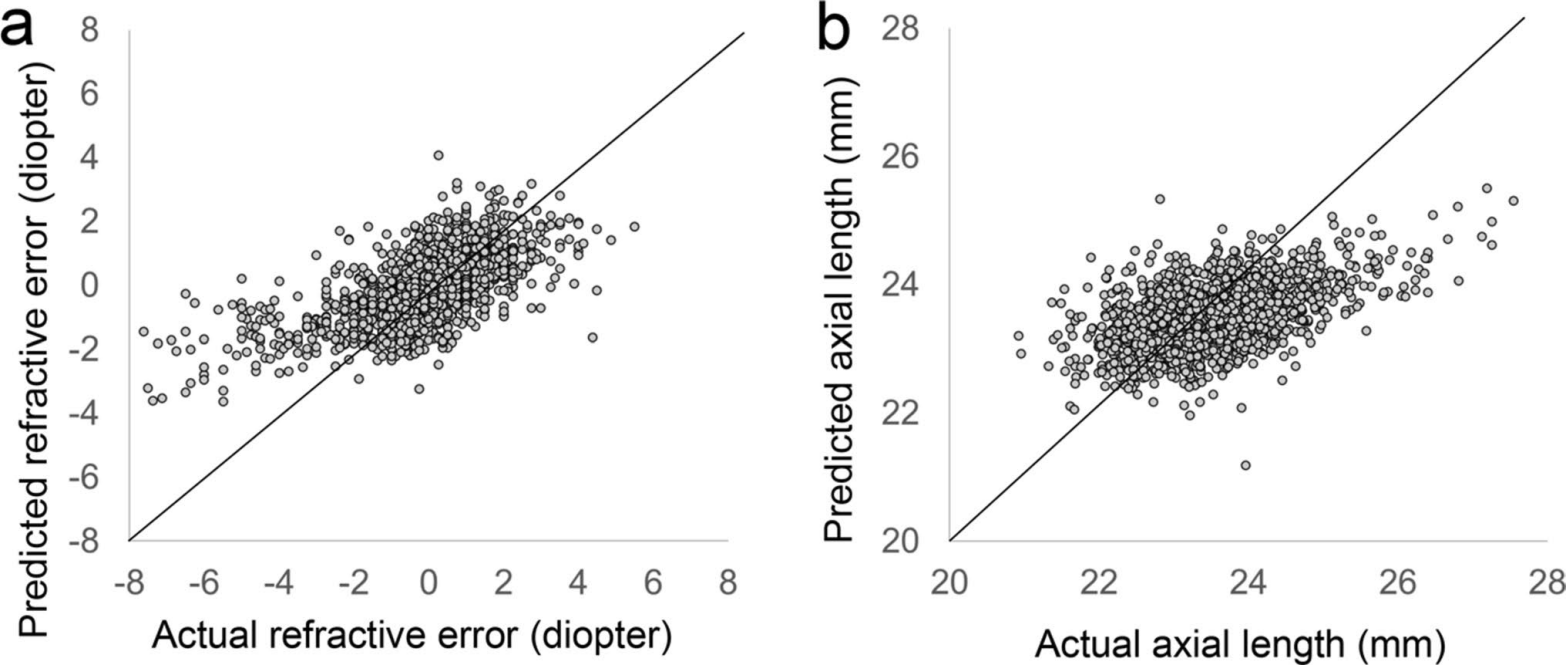
Scatter plot analysis between actual and predicted **a** refractive error and **b** axial length. The line represents actual = predicted values

The MAE for predicting the RE of participants was 0.91 diopter (standard deviation, SD: 0.87) (Table 2). The correlation coefficient between the actual and predicted RE was 0.63 (p<0.001) (Fig. 2a). The optimal model for RE obtained using LASSO regression and Pearson’s correlation coefficient between the actual RE and the fundus parameters is listed in Table 3. The MAE for predicting the AL of participants was 0.59 diopter (SD: 0.47) (Table 4). The correlation coefficient between actual and predicted AL was 0.51 (p<0.001) (Fig. 2b). The optimal model for AL obtained using LASSO regression and Pearson’s correlation coefficient between the actual AL and the fundus parameters is shown in Table 5.

**Table 2:** Prediction accuracy of spherical equivalent in this and previous studies

	This study (Regression analysis)	UK biobank [3] (Artificial intelligence)	AREDS [3] (Artificial intelligence)
Actual RE (diopter)	– 0.14±1.62	– 0.34±2.57	0.60±2.08
Predicted RE (diopter)	– 0.14±1.05	Not available	Not available
Mean absolute error (diopter)	0.91	0.56	0.91
Correlation coefficient	0.63	0.95	0.83

*RE* Refractive error

**Table 3:** Optimal model for the refractive error obtained using the LASSO binomial logistic regression and Pearson’s correlation between actual refractive error and fundus parameters

Fundus parameters	Coefficient of LASSO regression	Pearson’s correlation Coefficient	*p*-value of Pearson’s correlation
Papillomacular position (PMP)	0.055	0.084	0.001
Ovality ratio	0.650	0.019	0.448
Retinal artery angle (ST-RA, IT-RA)			
Supratemporal	0.010	0.155	< 0.001
Infratemporal	0.005	0.109	< 0.001
Retinal vein angle (ST-RV, IT-RV)			
Supratemporal	0.006	0.127	< 0.001
Infratemporal	0.008	0.126	< 0.001
Red intensity (R)			
Temporal	– 0.045	0.040	0.104
Supratemporal	0.014	0.157	< 0.001
Superior	N.S.	0.246	< 0.001
Supranasal	0.013	0.183	< 0.001
Nasal	– 0.049	0.057	0.022
Infranasal	0.029	0.247	< 0.001
Inferior	0.009	0.231	< 0.001
Infratemporal	– 0.016	0.089	< 0.001
Fovea	0.030	0.118	< 0.001
Green intensity (G)			
Temporal	– 0.007	0.054	0.029
Supratemporal	0.023	0.182	< 0.001
Superior	0.002	0.305	< 0.001
Supranasal	0.048	0.228	< 0.001
Nasal	N.S.	0.100	< 0.001
Infranasal	0.005	0.312	< 0.001
Inferior	N.S.	0.258	< 0.001
Infratemporal	0.022	0.090	< 0.001
Fovea	– 0.050	0.265	< 0.001
Blue intensity (B)			
Temporal	N.S.	– 0.095	< 0.001
Supratemporal	– 0.010	– 0.005	0.850
Superior	N.S.	0.137	< 0.001
Supranasal	– 0.034	0.078	0.002
Nasal	0.005	0.007	0.767
Infranasal	N.S.	0.160	< 0.001
Inferior	0.003	0.074	0.003
Infratemporal	0.009	– 0.083	0.001
Fovea	0.014	0.280	< 0.001
Tessellation fundus index (TFI)			
Temporal	16.855	0.066	0.008
Supratemporal	N.S.	0.053	0.032
Superior	– 4.305	– 0.022	0.363
Supranasal	0.794	– 0.003	0.901
Nasal	13.342	– 0.009	0.719
Infranasal	– 0.215	– 0.028	0.253
Inferior	N.S.	0.050	0.042
Infratemporal	3.679	0.089	< 0.001
Fovea	– 11.271	– 0.327	< 0.001

*N.S.* not selected, *ST-RA* supra temporal retinal artery angle, *IT-RA* infra temporal retinal artery angle, *ST-RV* supra temporal retinal vein angle, *IT-RV* infra temporal retinal vein angle

**Table 4:** Prediction accuracy of axial length in this and previous studies

	This study (Regression analysis)	Beijing Eye study 2011 [4] (Artificial intelligence)
Actual AL (mm)	23.50±0.88	23.29±1.17
Predicted AL (diopter)	23.50±0.48	Not available
Mean absolute error (diopter)	0.59	0.56
Correlation coefficient	0.51	0.77

*AL* Axial length

**Table 5:** Optimal model for the axial length obtained using the LASSO binomial logistic regression and Pearson’s correlation between actual axial length and fundus parameters

Fundus parameters	Coefficient of LASSO regression	Pearson’s correlation coefficient	*p*-value of Pearson’s correlation
Papillomacular position (PMP)	– 0.015	– 0.063	0.011
Ovality ratio	N.S.	0.031	0.203
Retinal artery angle (ST-RA, IT-RA)			
Supratemporal	– 0.006	– 0.138	< 0.001
Infratemporal	– 0.003	– 0.096	< 0.001
Retinal vein angle (ST-RV, IT-RV)			
Supratemporal	– 0.002	– 0.098	< 0.001
Infratemporal	– 0.006	– 0.133	< 0.001
Red intensity (R)			
Temporal	0.007	– 0.007	0.778
Supratemporal	N.S.	– 0.087	< 0.001
Superior	N.S.	– 0.145	< 0.001
Supranasal	N.S.	– 0.078	0.001
Nasal	0.013	0.033	0.187
Infranasal	– 0.006	– 0.129	< 0.001
Inferior	N.S.	– 0.119	< 0.001
Infratemporal	0.003	– 0.030	0.218
Fovea	N.S.	– 0.053	0.031
Green intensity (G)			
Temporal	0.009	– 0.116	< 0.001
Supratemporal	– 0.012	– 0.187	< 0.001
Superior	– 0.006	– 0.251	< 0.001
Supranasal	– 0.022	– 0.152	< 0.001
Nasal	N.S.	– 0.017	0.481
Infranasal	– 0.013	– 0.223	< 0.001
Inferior	– 0.009	– 0.237	< 0.001
Infratemporal	– 0.002	– 0.132	< 0.001
Fovea	N.S.	– 0.170	
Blue intensity (B)			< 0.001
Temporal	N.S.	– 0.047	0.056
Supratemporal	N.S.	– 0.082	0.001
Superior	0.007	– 0.155	0.001
Supranasal	0.001	– 0.060	0.015
Nasal	0.026	0.040	0.104
Infranasal	N.S.	– 0.143	0.001
Inferior	N.S.	– 0.157	0.001
Infratemporal	N.S.	– 0.048	0.050
Fovea	N.S.	– 0.179	0.001
Tessellation fundus index (TFI)			
Temporal	N.S.	0.097	< 0.001
Supratemporal	N.S.	0.094	< 0.001
Superior	N.S.	0.125	< 0.001
Supranasal	N.S.	0.070	0.004
Nasal	– 4.278	0.040	0.001
Infranasal	N.S.	0.104	< 0.001
Inferior	2.972	0.123	< 0.001
Infratemporal	N.S.	0.083	0.001
Fovea	1.440	0.225	< 0.001

*N.S.* not selected, *ST-RA* supra temporal retinal artery angle, *IT-RA* infra temporal retinal artery angle, *ST-RV* supra temporal retinal vein angle, *IT-RV* infra temporal retinal vein angle

**Fig. 3:**
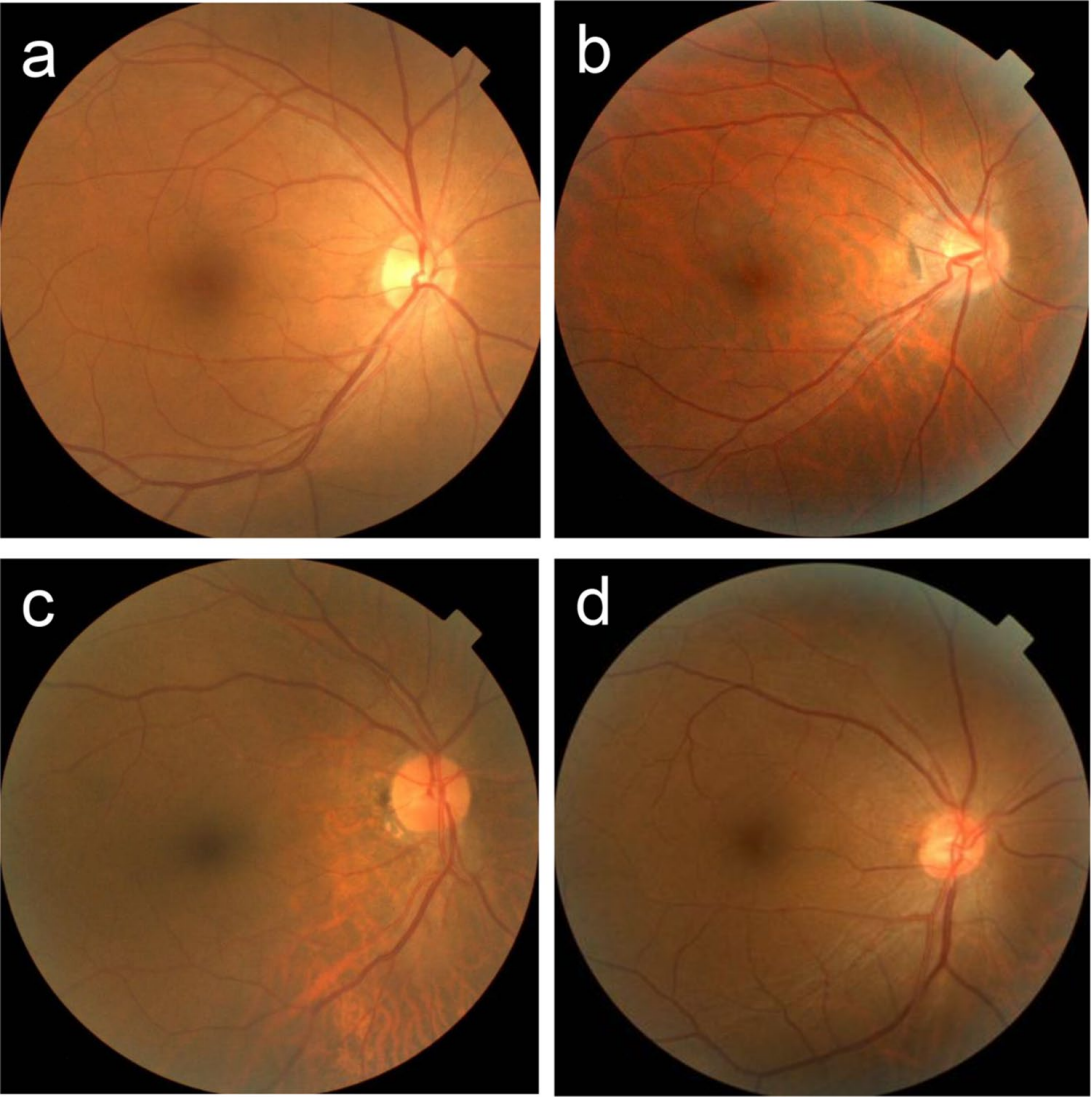
Typical and atypical fundus. The typical fundi of **a** a shorter AL eye (actual AL: 22.48 mm, predicted AL: 23.46 mm) and **b** a longer AL eye (actual AL: 25.12 mm, predicted AL: 25.16 mm). Atypical fundi of **c** a shorter AL eye with myopic fundus changes (actual AL: 20.95 mm, predicted AL: 22.93 mm) and **d** a longer AL eye with few myopic fundus changes (actual AL: 25.37 mm, predicted AL: 23.81 mm)

Eyes with a longer AL had narrower temporal vessel angles, weaker green intensities, stronger blue intensities, and stronger tessellation of the fundus color (p<0.001). The typical fundi of (**a**) a shorter AL eye (actual AL: 22.48 mm, predicted AL: 23.46 mm) and (**b**) a longer AL eye (actual AL: 25.12 mm, predicted AL: 25.16 mm) are shown in Fig. 3. Some eyes with a discrepancy between the AL and fundus myopic changes were observed in this study. These atypical fundi of (**c**) a shorter AL eye with myopic fundus changes (actual AL: 20.95 mm, predicted AL: 22.93 mm) and (**d**) a longer AL eye with few myopic fundus changes (actual AL: 25.37 mm, predicted AL: 23.81 mm) are shown in Fig. 3.

## Discussion

The accuracy of RE and AL prediction using LASSO regression in the Kumejima population study was lower than that observed in a previous report on deep-learning AI, which was also a cross-sectional study with participants aged >40 years. In general, the prediction accuracy of deep-learning AI outperformed that of regression analysis. For example, in sex determination, deep-learning AI has a 97% accuracy rate compared to 80.4% for regression analysis [2, 20]. The regression analysis was lower than the AI for RE and AL prediction because the AI calculates more fundus features than the 42 parameters included in this study. Similar studies measuring more detailed parameters are required. Another reason is the distribution of the participants. Figure 2 shows that the discrepancy between the actual and predicted values was larger in the myopic eyes. The standard deviations of actual RE of participants and the correlation coefficients between actual and predicted RE were 2.57, 2.08, and 1.62, and 0.95, 0.83, and 0.63 in the UK biobank, AREDS [3], and in this study, respectively. This indicates that the distribution of RE in the Kumejima study was narrower than in the AI studies. The Kumejima population study had fewer myopic subjects compared to other Japanese population studies, such as the Tajimi study [28]. This narrow distribution in the Kumejima study reduced the accuracy of predictions, especially for eyes with high myopia. A population with a wider distribution of RE may improve the prediction accuracy of the regression analysis. Furthermore, this study targeted subjects aged 40 and over and excluded young generations, who have a high incidence of myopia, contributing to the low prediction accuracy.

Attention heat maps are a method for analyzing where the AI focuses on an image. Attention heat map analysis in a previous AI study highlighted the macula and diffuse signals, such as retinal vessels and various parts of the fundus [3]. It is not known how the AI processes information from the macular areas and vessels. Therefore, it is unclear whether it can be used as a reference for positioning or color information of the macular area. Although we measured macular color and retinal vessel angles in this study, the prediction accuracy of RE and AL using regression analysis was lower than AI alone. This may be due to a more detailed quantification of blood vessel trajectories and diameters or analysis of several locations of the macula. Another possibility is that AI uses more complex calculations, whereas linear regression assumes a continuous linear correlation, which may be the reason for the lower prediction accuracy of linear regression compared with AI.

The cause of the decrease in the prediction accuracy in both the AI and this study was the atypical fundus, shown in Fig. 3c and d. There were eyes with large myopic changes despite a short axial length (AL) and eyes with few myopic changes despite a long AL. An earlier study shows large variations in AL at birth [29]. Thus, a long AL at birth does not necessarily mean that AL increases after attaining full growth. More specifically, even though the two eyes have the same AL as adults but differ at birth, the degree of elongation must have been different between these eyes during the growth period. Additionally, focal tessellations such as the inferior, peripapillary, and posterior pole areas are common in normal eyes [30]. A previous study shows that the AL of the eyes with peripapillary tessellation was shorter than of the eyes with posterior pole tessellation [17]. These paradoxical fundi with focal tessellation (Fig. 3c) may have affected the results of this study.

Pearson's correlation coefficient shows that the vessel angles got shallower in the longer AL and smaller RE groups. In myopic eyes, the temporal retinal artery and vein are closer to the fovea, caused by excessive axial elongation (oval change) during school age [31, 32]. Additionally, peripapillary tessellation tended to be stronger in eyes with a longer AL. Reportedly, the choroid [33] becomes thinner with longer AL, and the thinner the choroid, the stronger is the tessellation [15]. These findings are consistent with those of the present study. However, no significant correlation was observed between peripapillary tessellation and RE. This discrepancy is probably because AL directly represents eye elongation, whereas RE is the sum of AL and refraction of the cornea and lens.

In this study, compared to the results of deep-learning AI, the prediction accuracy of RE was lower than of AL. Compared to AL, which directly represents fundus changes due to eyeball elongation, RE includes information on the lens and cornea in addition to AL. AI may also be detecting the effects of the cornea and lens on the fundus from fundus photographs. Future research is needed to investigate the effects of lens thickness and corneal curvature on fundus photography independently of AL.

Pearson's correlation coefficient shows that the red, green, and blue intensities in almost the entire peripapillary area tended to weaken in the longer AL and smaller RE groups. This implies that the intensities of red, green, and blue decreased as the distance (AL) increased. The intensity of light from a point source is inversely proportional to the square of the distance from the source. Therefore, an object twice as far away receives only one-quarter of its energy [34]. Another property of light is its cosine law [35]. When illuminating a surface at a certain distance from a light source, the illuminance is brightest when the surface is perpendicular to the direction of light travel and becomes darker when the surface is tilted. The illuminance is proportional to the cosine of the angle of incidence of the light. In eyes with a longer AL, the curvature of the peripapillary eyewall becomes steeper, causing optic disc tilting. Therefore, the intensity of the peripapillary area decreases in eyes with long axial lengths. These “inverse-square law” and “cosine law” may affect the intensities of the fundus photographs.

Because the fundus parameters interact with each other, the results of the LASSO regression differed in part from the Pearson’s correlation results. Our previous studies on sex determination using ridge regression analysis [20] and attention heatmap analysis in an AI study [2] revealed that a comprehensive judgment using many factors can achieve high accuracy. It is challenging for humans to make comprehensive judgments using many factors; however, multiple regression analysis can help identify crucial factors associated with AL- and RE-related fundus changes.

This study has some limitations. First, this was a cross-sectional study conducted in individuals over 40 years of age with age-related changes, similar to previously reported AI studies. Hence, future longitudinal cohort studies in young adults with a narrow age distribution are required to investigate myopic changes in the fundus more accurately. Secondly, this was a Japanese epidemiological survey of the southern islands. The results may differ in other ethnic groups. This study used the normal eye database from the Kumejima population study and excluded 14 eyes with high myopia (RE < -8D) or high hyperopia (RE > +5D), which may have resulted in low prediction accuracy.

In conclusion, LASSO regression analyses using fundus parameters can predict AL and RE in the Kumejima population to a lesser extent than deep-learning AI. RE- or AL-related changes in fundus photography included vessel angles and peripapillary color intensity. RE- or AL-related changes in the fundus may aid in understanding the mechanisms of myopia and fundus diseases such as glaucoma.
